# Differentiation of Saccadic Eye Movement Signals

**DOI:** 10.3390/s21155021

**Published:** 2021-07-24

**Authors:** Roberto A. Becerra-García, Rodolfo García-Bermúdez, Gonzalo Joya

**Affiliations:** 1 Departamento de Tecnología Electrónica, Universidad de Málaga, CEI Andalucía Tech, 29071 Málaga, Spain; idertator@uma.es (R.A.B.-G.); gjoya@uma.es (G.J.); 2 Departamento de Informática y Electrónica, Universidad Técnica de Manabí, Portoviejo 130105, Ecuador; 3 Instituto Universitario de Investigación en Telecomunicación (TELMA), Universidad de Málaga, CEI Andalucía Tech, E.T.S.I. Telecomunicación, 29071 Málaga, Spain

**Keywords:** numerical differentiation, electrooculograms, saccades identification, saccades biomarkers computing

## Abstract

Saccadic electrooculograms are discrete biosignals that contain the instantaneous angular position of the human eyes as a response to saccadic visual stimuli. These signals are essential to monitor and evaluate several neurological diseases, such as Spinocerebellar Ataxia type 2 (SCA2). For this, biomarkers such as peak velocity, latency and duration are computed. To compute these biomarkers, we need to obtain the velocity profile of the signals using numerical differentiation methods. These methods are affected by the noise present in the electrooculograms, specially in subjects that suffer neurological diseases. This noise complicates the comparison of the differentiation methods using real saccadic signals because of the impossibility of establishing exact saccadic onset and offset points. In this work, we evaluate 16 differentiation methods by the design of an experiment that uses synthetic saccadic electrooculograms generated from parametric models of both healthy subjects and subjects suffering from Spinocerebellar Ataxia type 2 (SCA2). For these synthetic electrooculograms the exact velocity profile is known, hence we can use them as a reference for comparison and error computing for the tasks of saccade identification and saccade biomarker computing. Finally, we identify the best fitting method or methods for each evaluated task.

## 1. Introduction

Eye movements are those performed by the eyes as a response to some environmental stimulus. For neurologists, the study of the control of eye movements presents an opportunity to understand the human brain [1]. Moreover, these movements have a very useful role because they can identify disfunctions caused by several neurological diseases such as Spinocerebellar Ataxia type 2 (SCA2). Furthermore, pursuit and saccadic movements are necessary to track objects in motion and provide a tool to explore neural functions [2].

Electrooculography (EOG) is a technique used to capture eye movements in clinical research. It is based on the measurement of potential generated in the retina–cornea area of the ocular system [3]. This technique was introduced by Fenn and Hursh in 1934 and uses superficial electrodes around the skin of the eyes. The resulting potential signal is called an electrooculogram and can be translated later into an angular movement signal using calibration methods.

Saccades are abrupt eye movements performed to move images of objects of interest to the fovea. Diseases such as Spinocerebellar Ataxias affect the performing of the saccadic system. For example, SCA2 provokes slowdowns in the saccadic movements [4]. Numerically, a saccade is a vector of contiguous eye positions that belong to an electrooculogram (measured in angular degrees).

The velocity profile of an electrooculogram is a vector of instantaneous velocity points associated to the position vector of the electrooculogram. Obtaining this velocity profile is one step of saccade identification algorithms. For instance, one of the classic papers in saccade identification recommends use always of the velocity as the criteria to identify onset and offset saccade points [5]. This profile allows us to compute relevant biomarkers such as peak velocity, latency and duration. We compute the instantaneous velocity profile of an electrooculogram by the differentiation of its values.

Given the discrete nature of the electrooculograms, there is a requirement to use numerical differentiation methods to obtain the velocity profiles. These methods always introduce a level of noise to their output (velocity profile), even when the position profile is noise-free.

**Figure 1:**
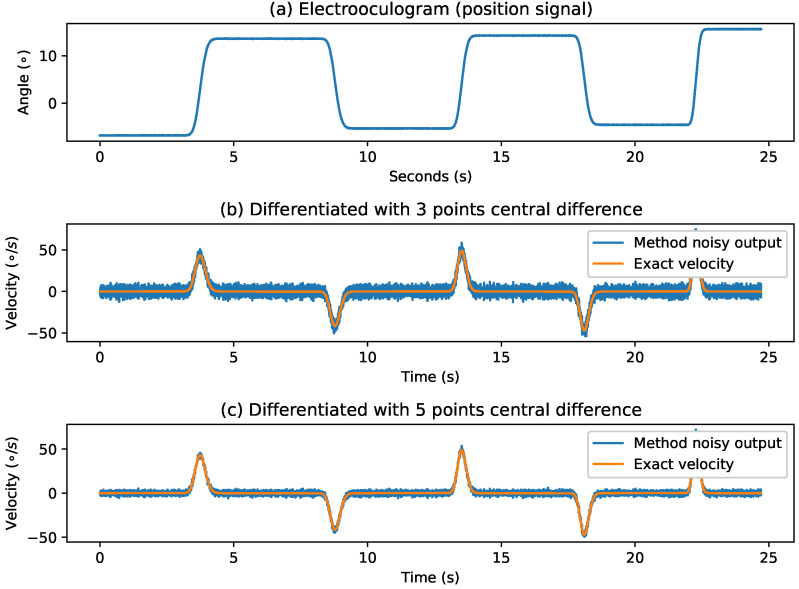
An example of an electrooculogram differentiated using the central difference of 3 and 5 points.

Thus, Figure 1 shows how, from an electrooculogram with almost no noise, the output of the differentiation method presents a high level of noise. Figure 1a shows a very clean position signal of a saccadic electrooculogram. In Figure 1b,c we show noisy velocity profiles computed from the position signal using a central difference of three and five points, respectively. The noise of the output affects the identification of the position of the onset and offset points of a saccade. This situation leads to errors in the calculation process of important biomarkers such as maximum velocity, latency and duration of saccades.

In the literature reviewed we found four numerical differentiation method families based on different mathematical approaches: Central Difference, Lanczos, Super Lanczos and Smooth Noise Robust. Methods such as Central Difference and Lanczos have been used to differentiate electrooculograms. However, for the rest of the methods we found no usage for these signals. Is very interesting to evaluate how to perform methods such as Super Lanczos and Smooth Noise Robust for our specific tasks.

Researchers use central difference methods with acceptable results when the signal is noise-free [6,7]. However, Figure 1 shows the undesired effects of the noise in the differentiation output of these methods. Note how a minor noise in the movement signals produces a very noisy differentiated signal. in addition, filtering the signal before differentiation does not improve the output of the process.

The signals captured using devices like electronystagmographers or eye trackers produce electrooculograms which may include several noises such as tremors (biological), power line noise, digitalization noise, and others. Using these position signals we can not obtain the associated exact velocity profile because of the included noise, hence is impossible to create a framework to evaluate the performance of the numerical differentiators.

The goal of this work is to compare numerical differentiation methods available in the literature for its application in saccadic identification and biomarker extraction tasks. This comparison must be based on quantitative values of the errors introduced in the referred tasks. To measure the performance of each method, we will use a set of synthetic saccadic signals at different amplitudes and subject statuses (healthy or sick with SCA2) with added noise. These signals allow knowledge of the exact values of the errors introduced because the exact values of the biomarkers associated with these signals are also known.

In summary, we consider that this work presents two major contributions: (a) we find the best method to differentiate saccadic electrooculograms, (b) we provide the implementation of these methods for free in a GitHub repository.

The rest of this paper is organized as follows: In the Material and Methods section we describe the experiment designed to compare the differentiation methods. The Discussion section shows an analysis of the designed experiment results. Finally, the Conclusions section summarizes the main ideas and findings of this work.

## 2. Material and Methods

### 2.1. Numerical Differentiation

The derivative of a function *f* in $x_{0}$ is defined in the Equation (1) [8]:

(1) $$f^{\prime }\left(x_{0}\right)=\lim_{h\to 0}=\frac{f\left(x_{0}+h\right)-f\left(x_{0}\right)}{h}$$

Using Lagrange’s interpolation polynomials, we can develop several differentiation methods based on central difference. Equation (2) represents the general form of the central difference methods.

(2) $$f^{\prime }\left(x_{0}\right)\approx \frac{1}{h}\sum_{k=1}^{(N-1)/2}a_{k}\left(f_{k}-f_{-k}\right)$$

In this equation, $x_{0}$ is the point where the instant velocity is calculated, $f_{\pm k}$ represents $f(x_{0}\pm kh)$, *h* is the time interval between samples, and $a_{k}$ are the parameters to be determined.

The 3-point central difference was proposed by Bahill and McDonald in [6] and Niemenlehto in [7] to differentiate eye movement signals. This last method has to be used with a low-pass filter to obtain reliable results [9].

Inchingolo and Spanio proposed in [10] an algorithm to calculate the velocity profile of eye movement signals that is a particularization of the nine-point central difference. This method is described by Equation (3) where $f_{s}$ is the sampling frequency. In the particular case of signals sampled at 200Hz, authors found that the best coefficients are $a_{1}=0.8024$, $a_{2}=-0.2022$, $a_{3}=0.03904$ and $a_{4}=-0.003732$.

(3) $$f^{\prime }\left(x_{0}\right)=f_{s}\sum_{k=1}^{4}a_{k}\left(f_{k}-f_{-k}\right)$$

Analog to central difference methods, the Lanczos methods have been developed as a particular set of Savitzky–Golay [11] differentiation filters. The fundamental difference regarding their predecessors is that they use curve fitting strategies instead of interpolation, making them more noise-robust. Lanczos differentiators works as follows: for a fixed *h* step and sample f(x) at odd *N* points around a central point $x_{0}$ we construct the polynomial shown in Equation (4) minimizing the cost function shown in Equation (5) with respect to unknown coefficients $a_{j}$ [12].

(4) $$P_{M}\left(x\right)=\sum_{j=0}^{M}a_{j}x^{j}$$

(5) $$Z=\sum_{k=-\left(N-1\right)/2}^{\left(N-1\right)/2}{\left(f_{k}-P_{M}\left(x_{k}\right)\right)}^{2}$$

After the polynomial is computed, $f^{\prime }\left(x_{0}\right)$ can be estimated as:

(6) $$f^{\prime }\left(x_{0}\right)=P_{M}^{\prime }\left(x_{0}\right)$$

We call Lanczos differentiators to the filters built using M=2 and Super Lanczos when M=4.

One last family to be considered is the Smooth Noise-Robust methods [12]. They make up a variation of Lanczos family, and they are described by Equations (7) and (8)

(7) $$f^{\prime }\left(x_{0}\right)\approx \frac{1}{h}\sum_{k=1}^{M}c_{k}\left(f_{k}-f_{-k}\right)$$

(8) ck=122m+12mm−k+1−2mm−k−1,m=N−32,M=N−12

where *N* is the filter length, as in the previous equations.

### 2.2. Experiment Design

With the goal to choose the best fitting numerical differentiation method for saccadic electrooculograms, an experiment was designed. In this experiment we compare 16 methods belonging to four families: Central Difference (CD3, CD5, CD7, CD9), Lanczos (L5, L7, L9, L11), Super-Lanczos (SL7, SL9, SL11), Smooth Noise-Robust (SNR5, SNR7, SNR9, SNR11). Each number attached as a suffix to the method name means the length of the corresponding filter.

Our experiment compares the performance of the differentiation methods using 4 different metrics:

- Mean Square Error (MSE) between the output of the method as approximated signal and the synthetic real velocity profiles as the exact signal.
- Misidentified saccades.
- Over-identified saccades.
- Absolute error introduced in the biomarkers values.

The MSE is computed from the output of the differentiation methods with respect to the exact velocity profile of synthesized signals. This metric gives a quantitative value, which describes similarity or, in contrast, the level of error/distortion between the signals. Formally, the operation is defined as follows: given two discrete signals *x* and *y* of finite length, $x=\{x_{i}|i=1,2,\ldots ,n\}$ and $y=\{y_{i}|i=1,2,\ldots ,n\}$, where *n* is the number of samples of the signals, and $x_{i}$ and $y_{i}$ are the value of the i-th samples of *x* and *y*, respectively, the Mean Square Error between both signals is described in Equation (9).

(9) $$MSE\left(x,y\right)=\frac{1}{n}\sum_{i=1}^{n}{\left(x_{i}-y_{i}\right)}^{2}$$

To obtain saccadic biomarkers, first we need to identify the saccades. We can evaluate the performance of the differentiation methods by obtaining how many saccades are misidentified or over-identified using a simple velocity threshold algorithm with the output (velocity profile) of each method. For this algorithm we are going to use the same velocity threshold employed to generate the synthetic signals as onset and offset thresholds.

There are many biomarkers used to study SCA2. Among the most common and relevant are the Latency, Duration and Peak Velocity. Latency is the time between the start of the visual stimulus and the response of the subject. The duration of the saccade is the time between its start and its end. The Peak Velocity is, from our experience, the most important biomarker to diagnose SCA2 and is the maximal velocity reached during the saccade.

The designed experiment is structured as follows:

1. Generate synthetic saccadic records using characteristics parameters obtained from electrooculograms of healthy and SCA2-sick subjects. We obtain the exact velocity profile (EVP) from which the saccadic records are generated.
2. Apply each differentiation method to the synthetic electrooculograms with noise added, resulting in the approximated velocity profiles (AVP).
3. For each AVP:
  (a) Compute the MSE between the EVP and AVP. Analyze the results and drop methods with significantly poor performance.
  (b) Identify saccades using the AVP and compare them against the exact saccades identified using the EVP. We compare the performance of the identification process using misidentified and over-identified saccade metrics. All the saccades correctly identified using the AVP are defined as AS and their corresponding exact counterparts identified using the EVP as defined as ES.
  (c) For each AS and their associated ES we compute the biomarkers peak velocity, latency and duration. For each pair (ES, AS) and for each biomarker, we compute the error using the absolute value of biomarker (ES)– biomarker (AS).
4. Analyze statistically the results yielded by the previous step and determine which methods to use for the different tasks in processing saccadic eye movements.

Regarding step 4 of our experiment, a comparative analysis using the Friedman statistical test was performed [13]. This is a non-parametric statistical test equivalent to Analysis of Variance (ANOVA) with repeated measures, which determines if there are significant differences between the results of a set of methods over the same datasets. Applying the Friedman test to the result yielded by each biomarker allows the determination that for each of the biomarkers there are significant differences among their means.

Now, to determine which of the methods are fit to compute each of the saccadic biomarkers, we applied a post-hoc Wilcoxon signed-ranked test pairing the method with lower error with the rest [14]. Each of the tests determines if there are significant differences between the pairs of biomarker means.

### 2.3. Building Synthetic Saccadic Signals Dataset

The set of saccade signals employed for the comparison was generated synthetically using the method described by Coughlin in [15]. This algorithm follows an inverse process regarding the natural generation of the signals: first the velocity profiles are generated and then they are integrated to obtain the position profiles. The characteristic parameters used to generate the synthetic velocity profiles were maximum velocity, latency and duration obtained from a statistical analysis performed on healthy and SCA2-sick subjects.

To make the signals as real as possible, a set of noises found in real electrooculograms were added. Specifically, sinusoidal interference of 60 Hz simulating noise introduced by the industrial network, white noise, which has a uniform spectral distribution and color noise between 3 and 7 Hz. The color noise was found when performing the spectral analysis of records of people with the disease.

With the goal to obtain reliable statistic results, a set of 120 signals are generated and distributed, as shown in Table 1. Each of these signals contains a set of 20 saccades, making 2400 saccades in the full dataset. Here the saccades are generated from stimulation angles of 20, 30 and 60 degrees as found in real clinical electrooculograms.

The signals were generated with a sampling frequency equal to 1000 Hz. However, to mimic the characteristics of the real signals, we need to re-sample the synthetic signals to a sampling frequency of 200 Hz. To make this we use the function *decimate* (https://docs.scipy.org/doc/scipy/reference/generated/scipy.signal.decimate.html, accessed on 23 July 2021) of the SciPy library [16]. This function downsamples the signal after applying an 8th Order Chebyshev antialiasing filter. Is important to note that all the calculations of the experiment are performed using the 200 Hz re-sampled signals.

### 2.4. Saccade Identification Algorithm

The previous step to biomarker computing is identifying the saccades from which they are going to be extracted. There are several methods to identify saccades in eye movement recordings; we are going to use a simple velocity based saccade identification detailed by Algorithm 1. In this algorithm, we use the output of the differentiation methods as *V*. For the occurrence threshold $O_{t}$ we select the used value to generate the synthesized signals, and to set the onset and offset points of the saccades we use $P_{t}=20^{\circ }$/s [17,18]. The step *h* is equal to 0.05 s because the signals are sampled using a 200 Hz frequency.
**Algorithm 1:** Velocity threshold saccade identification algorithm
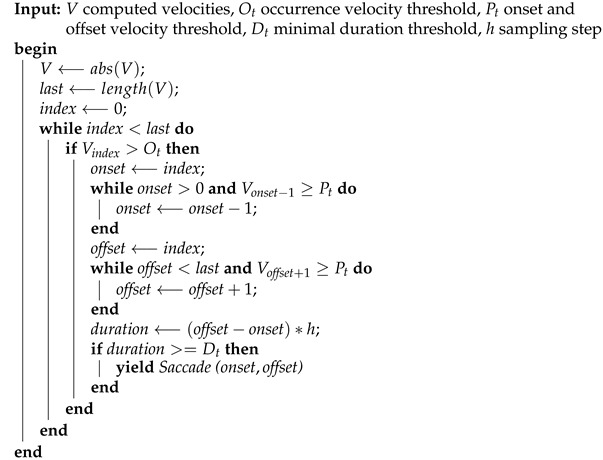


## 3. Results and Discussion

### 3.1. Error of the Waverform of the Output Filter

**Figure 2:**
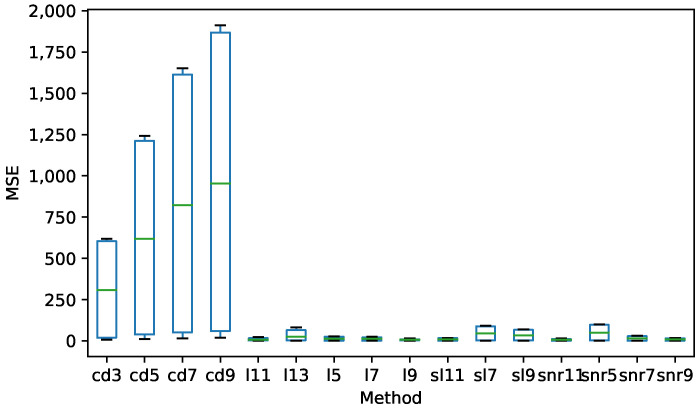
Errors introduced by differentiation methods measured using the Mean Squared Error (MSE).

To compare the signal waveform errors introduced by the differentiation algorithm, we build the box plot shown in Figure 2. In this plot we can notice how the central difference methods introduce errors higher than the rest by several orders of magnitude.

**Figure 3:**
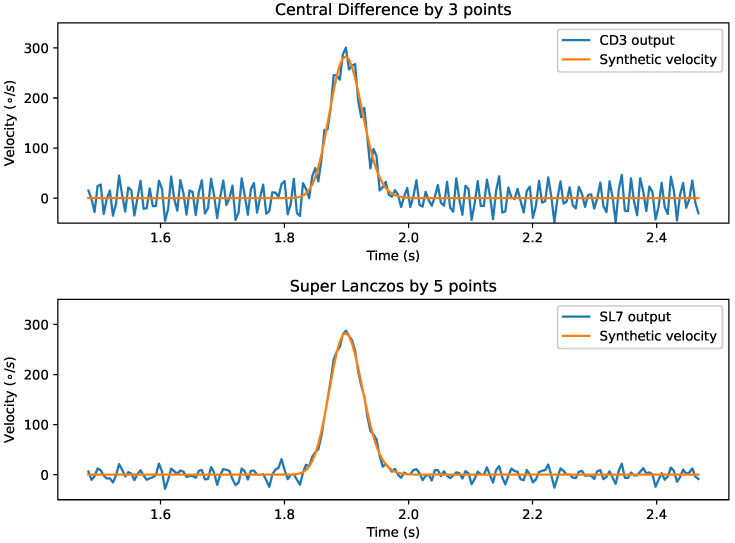
Comparison between the best central difference method and the worst of the rest of the families. The signal is from a healthy synthetic signal with saccades of $20^{\circ }$.

Figure 3 shows an example of the poor performance of the central difference method against the worst performing of the rest. In the figure, the error of the peak velocity introduced by the noise is also noticeable. Moreover, this noise is present in the regions near the points of saccade onset and offset, hindering the correct identification of saccade. This low performance can be explained because of the instability inherent to numerical differentiation methods added to the principle of interpretation used by the central difference methods [8]. For these reasons, these methods will be dropped to further analyze and focus our efforts on more adequate candidates for the tasks.

### 3.2. Saccade Identification Errors

**Figure 4:**
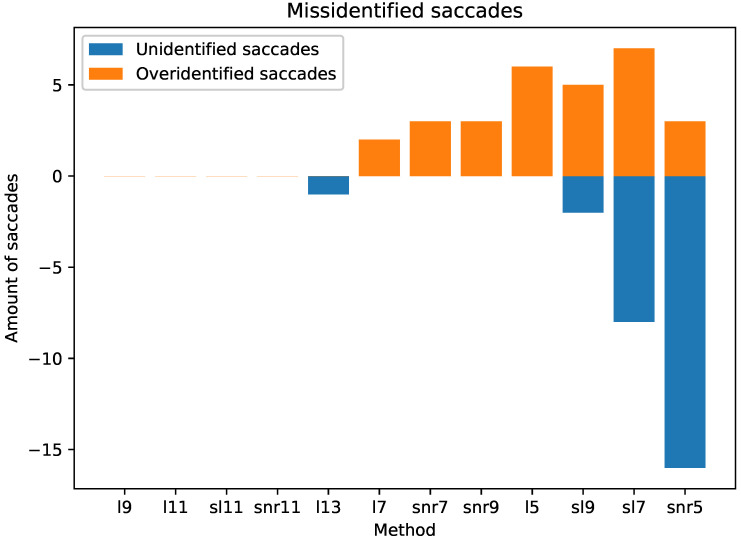
Number of misidentified saccades by the different methods.

We use two metrics to measure the performance of the identification algorithms: unidentified saccades and over-identified saccades. Unidentified saccades are the amount of saccades that should be identified by the algorithm and were not. Over-identified saccades are the amount of saccades detected as false positives by the algorithm. Figure 4 shows the errors introduced by using the output of the differentiation method as the output for the identification algorithm.

The performance of the analyzed methods is very satisfactory. From 2400 saccades, the worst method (snr5) misidentified only 20 saccades, less than 1%. Furthermore, more importantly, four methods show a perfect score. It is also noticeable that all the 11-point methods are in the set of perfect score.

### 3.3. Biomarkers Calculations

The goal of the research regarding eye movements is to extract relevant knowledge which allows diagnosis and following of neurological diseases. This relevant knowledge is presented very often as biomarkers, hence the importance of their computing method. In this work, we analyze how the differentiation methods impact in the values of the relevant saccadic biomarkers to the research of the SCA2 such as Peak Velocity, Latency and Duration.

In Table 2 we show the results obtained by the use of the proposed differentiation methods. Applying the Friedman test, we detected significant differences in all of the biomarkers’ errors. Consequently, to identify the set of the fittest methods for the task, we pair the method with the lower error with each one of the rest by applying the Wilcoxon post-hoc test. The methods highlighted in bold belong to a cluster of methods in which the null hypothesis of Wilcoxon was accepted, meaning that the errors introduced by these methods have the same distribution.

Table 2 shows that for the saccadic peak velocity, the Super Lanczos Method with 11 points is the most fit for the task. This could mean that sl11 can maintain the better waveform of the differentiated signal around the point of maximal velocity (middle of the saccade). Further study is required.

Regarding saccadic latency, Table 2 shows three methods with the best performance: Smooth Noise Robust with 11 points, Super Lanczos with 11 points and Smooth Noise Robust with 9 points. These results are related to how well the saccade onsets are positioned, explaining how these methods affect the samples near to the start of the saccade.

In the case of saccadic duration, the Lanczos with 11 and 9 points have the best performance. Like the saccadic duration they are affected by the position of the saccadic onset, but are also affected by the saccadic offset position. The errors of the duration can be explained by the performance of the algorithms around the samples near the start and finish of the saccade.

**Figure 5:**
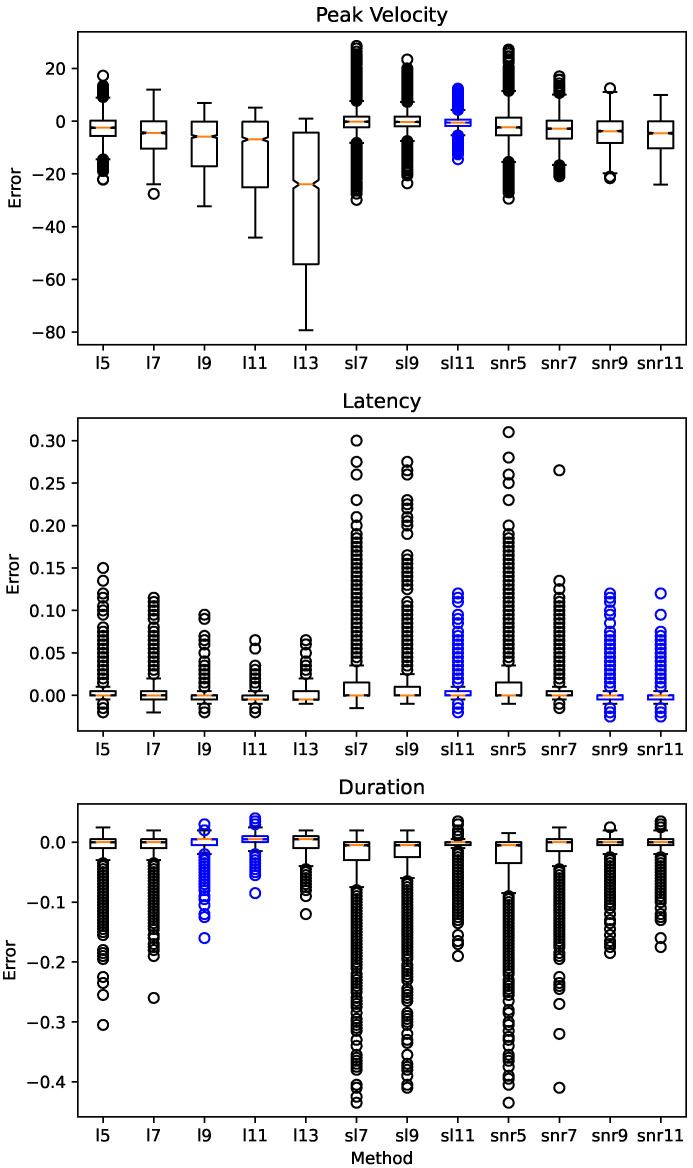
Biomarker computing errors box plot by method. Methods highlighted in blue for each task are those that show no significant difference with the first ranked using Wilcoxon post-hoc method.

Figure 5 shows the analyzed performance described in previous paragraphs in a more visual form.

Is interesting to notice how the best methods nominally always have 11 points. This could mean that 11 is the right size for the differentiation filters applied to signals with 200 Hz of sampling rate, or at least with the characteristics of saccadic signals similar to the ones synthesized in this work. To confirm this theory, more study is required in this regard.

## 4. Conclusions

In this paper we evaluated 16 numerical differentiation methods of 4 different families: Central Difference (cd), Lanczos (l), Super Lanczos (sl) and Smooth Noise Robust (snr) for saccadic signals differentiation of subjects suffering SCA2. First, we presented a review of the methods traditionally used for our specific task and others used in other areas of knowledge. We designed an experiment to compare the methods numerically using quality and error metrics.

Our first conclusion from our experiment is that the central difference methods are not adequate for our specific task. The level of noise introduced by these sets of methods hinders the further processing of the signals. For the saccade identification task, all the methods perform reasonably well, with the methods l9, l11, sl11 and snr11 obtaining perfect score.

For each saccadic biomarker included in our study, the experiment results in a unique set of methods fit to compute each one of them. With saccadic peak velocity, we recommend using the sl11 method. For saccadic latency computation we recommend the use of these methods: snr11, sl11, snr9. For saccadic duration you can use the l11 or the l9 methods.

Is important to remark that some high performing methods like sl11, snr9 and snr11 were not used previously with electrooculograms, being a key contribution of our paper.

## Figures and Tables

**Table 1:** Record distribution per subject status and angle.

Class	$20^{\circ }$	$30^{\circ }$	$60^{\circ }$	Total
*Healthy*	20	20	20	60
*SCA2-Sick*	20	20	20	60
*Total*	40	40	40	120

**Table 2:** Errors introduced by differentiation methods in saccadic biomarker computing. Methods highlighted in blue for each task are those that show no significant difference with the first ranked using the Wilcoxon post-hoc method.

Rank	Peak Velocity (${}^{\circ }/s$)		Latency (*s*)		Duration (*s*)	
	Method	Error ± Std	Method	Error ± Std	Method	Error ± Std
1	**sl11**	2.1375 ± 2.2142	**snr11**	0.0045 ± 0.0080	**l11**	0.0079 ± 0.0065
2	sl9	3.8759 ± 4.3384	l11	0.0045 ± 0.0042	snr11	0.0084 ± 0.0135
3	l5	4.1076 ± 3.4649	l9	0.0047 ± 0.0064	**l9**	0.0085 ± 0.0115
4	sl7	4.4216 ± 5.2121	**sl11**	0.0049 ± 0.0104	sl11	0.0098 ± 0.0186
5	snr7	4.5968 ± 3.9061	**snr9**	0.0051 ± 0.0098	snr9	0.0100 ± 0.0175
6	snr9	5.1840 ± 4.4540	l13	0.0065 ± 0.0048	l13	0.0129 ± 0.0094
7	snr5	5.7055 ± 5.3013	l7	0.0067 ± 0.0116	l7	0.0141 ± 0.0235
8	snr11	6.0861 ± 5.4302	l5	0.0069 ± 0.0138	l5	0.0143 ± 0.0266
9	l7	6.4026 ± 5.5485	snr7	0.0076 ± 0.0150	snr7	0.0161 ± 0.0299
10	l9	9.4111 ± 9.0850	sl9	0.0128 ± 0.0259	sl9	0.0274 ± 0.0510
11	l11	13.3196 ± 13.4722	sl7	0.0145 ± 0.0299	sl7	0.0314 ± 0.0581
12	l13	29.6168 ± 26.0764	snr5	0.0147 ± 0.0283	snr5	0.0324 ± 0.0568

## Data Availability

The data used in our research are synthetic signals generated using parameters computed from a population of real subjects. Is not possible to use these signals to identify any individual. All the raw data and experimental software routines are freely available at https://github.com/idertator/saccdiff (accessed on 23 July 2021). The software routines included is used to generate figures and tables used in the analysis of our research.

## Abbreviations

The following abbreviations are used in this manuscript:

ANOVA	Analysis of Variance
EOG	Electrooculography
MSE	Mean Square Error
SCA2	Spinocerebellar Ataxia type 2
